# The effect of 3*β*, 6*β*, 16*β*-trihydroxylup-20(29)-ene lupane compound isolated from *Combretum leprosum* Mart. on peripheral blood mononuclear cells

**DOI:** 10.1186/s12906-015-0948-1

**Published:** 2015-11-25

**Authors:** Fabianne Lacouth-Silva, Caroline V. Xavier, Sulamita da S. Setúbal, Adriana S. Pontes, Neriane M. Nery, Onassis Boeri de Castro, Carla F. C. Fernandes, Eduardo R. Honda, Fernando B. Zanchi, Leonardo A. Calderon, Rodrigo G. Stábeli, Andreimar M. Soares, Izaltina Silva-Jardim, Valdir A. Facundo, Juliana P. Zuliani

**Affiliations:** Laboratório de Imunologia Celular Aplicada à Saúde, Fundação Oswaldo Cruz, FIOCRUZ Rondônia, Rua da Beira, 7671 BR364, Km 3, 5, CEP 76812-245 Porto Velho, RO Brazil; Programa de Pós Graduação em Biologia Experimental, PGBIOEXP, Núcleo de Saúde, NUSAU, Universidade Federal de Rondônia, UNIR, Porto Velho, RO Brazil; Programa de Pós-Graduação em Biodiversidade e Biotecnlogia, Rede BIONORTE, Universidade Federal de Rondônia, UNIR, Porto Velho, RO Brazil; Centro de Pesquisa em Medicina Tropical, Porto Velho, RO Brazil; Laboratório Central de Saúde Publica de Rondonia, LACEN/RO, Porto Velho, RO Brazil; Centro de Estudos de Biomoléculas Aplicadas à Saúde (CEBio), Fundação Oswaldo Cruz, FIOCRUZ Rondônia e Departamento de Medicina, Universidade Federal de Rondônia, UNIR, Porto Velho, RO Brazil; Departamento de Ciências Biológicas, Universidade Estadual de Santa Cruz, UESC, Ilhéus, BA Brazil; Departamento de Química, Universidade Federal de Rondônia, UNIR, Porto Velho, RO Brazil

## Abstract

**Background:**

The *Combretum leprosum* Mart. plant, popularly known as mofumbo, is used in folk medicine for inflammation, pain and treatment of wounds. From this species, it is possible to isolate three triterpenes: (3*β*, 6*β*, 16*β*-trihydroxylup-20(29)-ene) called lupane, arjunolic acid and molic acid. In this study, through preclinical tests, the effect of lupane was evaluated on the cytotoxicity and on the ability to activate cellular function by the production of TNF-α, an inflammatory cytokine, and IL-10, an immuno regulatory cytokine was assessed. The effect of lupane on the enzymes topoisomerase I and II was also evaluated.

**Methods:**

For this reason, peripheral blood mononuclear cells (PBMCs) were obtained and cytotoxicity was assessed by the MTT method at three different times (1, 15 and 24 h), and different concentrations of lupane (0.3, 0.7, 1.5, 6, 3 and 12 μg/mL). The cell function was assessed by the production of TNF-α and IL-10 by PBMCs quantified by specific enzyme immunoassay (ELISA). The activity of topoisomerases was assayed by in vitro biological assays and *in silico* molecular docking.

**Results:**

The results obtained showed that lupane at concentrations below 1.5 μg/mL was not toxic to the cells. Moreover, lupane was not able to activate cellular functions and did not alter the production of IL-10 and TNF-α. Furthermore, the data showed that lupane has neither interfered in the action of topoisomerase I nor in the action of topoisomerase II.

**Conclusion:**

Based on preclinical results obtained in this study, we highlight that the compound studied (lupane) has moderate cytotoxicity, does not induce the production of TNF-α and IL-10, and does not act on human topoisomerases. Based on the results of this study and taking into consideration the reports about the anti-inflammatory and leishmanicidal activity of 3*β*, 6*β*, 16*β*-trihydroxylup-20(29)-ene, we suggest that this compound may serve as a biotechnological tool for the treatment of leishmaniasis in the future.

## Background

Plants are an important source of secondary metabolites that have been extensively used as bioactive components in therapeutically effective medicines for the treatment of various diseases [1, 2]. Before the advent of high-throughput screening and post-genomic technologies, the majority of drugs were natural products or inspired by a natural compound [3]. Among natural products, terpenes comprise a class of secondary plant metabolites which show high structural diversity and biological activity against important parasites including *Leishmania* sp [4].

Combretaceae is a large family of flowering plants that belongs to the Myrtales order with at least 600 species distributed throughout Asia, Africa and the Americas. Among its 18 genera, the *Combretum* genus is the most common, with approximately 370 species [5]. This genus is widely used in folk medicine for the treatment of hepatitis, malaria, respiratory infections and cancer in different parts of Asia and Africa [6]. In Brazil, the *Combretum leprosum* Mart species, popularly known as cipoaba, mofumbo or mufumbo, is found between the states of Piauí and Bahia [7, 8]. In this region, it is popularly used in the treatment of wounds in bleeding or as a sedative [9].

Facundo et al. [7] isolated the TTHL compound (3*β*, 6*β*, 16*β*-trihydroxylup-20(29)-ene) called lupane from *C. leprosum* leaves. Pietrovski et al. [10] evaluated the antinociceptive activity of the ethanolic extract obtained from *C. leprosum* flowers and the isolated compound TTHL, showing that the extract was able to inhibit nociception in different models, such as visceral hyperalgesia induced by acetic acid, hyperalgesia induced by heat (hot plate) and neurogenic and inflammatory hyperalgesia induced by formalin, capsaicin and glutamate. Additionally, extracts of *C. leprosum* fruits showed anti-snake venom properties by the inhibition of proteolytic activity and bleeding induced by *Bothrops jararacussu* and *B. jararaca* venoms, respectively [11].

The ethanolic extract of *C. leprosum* fruits and the isolated compound TTHL also showed the ability to inhibit the growth and formation of biofilms of *Streptococus mutans* and *S. mitis* [12] and antileishmanial activity against *L. amazonensis* promastigote forms [13].

The literature reports that some triterpenes have inhibitory activity against parasitic topoisomerase [14–16], and antileishmanial activity by inhibiting topoisomerase IB [17, 18]. Thus, the present study was developed to investigate the action of the triterpene lupane 3*β*, 6*β*, 16*β*-trihydroxylup-20(29)-ene isolated from *C. leprosum* flowers on human topoisomerases, as well as its effect on human cells, in order to contribute with the knowledge about the mechanism of action of lupane on human PBMC function.

## Methods

### Chemicals and reagents

RPMI-1640, L-glutamine, gentamicin, Histopaque 1077, DMSO and LPS (lipopolysaccharide) were purchased from Sigma (MO, USA). DuoSet ELISA Human IL-10 and DuoSet ELISA Human TNF-α/TNFSF1A were purchased from R&D Systems (MN, USA). Fetal bovine serum (FBS) was obtained from Cultilab (Sao Paulo, Brazil). Topoisomerase I and II kits were purchased from TopoGEN (FL, USA). All salts and reagents used were obtained from Merck (Darmstadt, Germany) with low endotoxin or endotoxin-free grades.

### Lupane

**Fig. 1:**
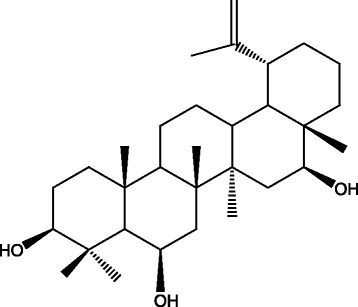
Molecular structure of triterpene lupane 3*β*, 6*β*, 16*β*-trihydroxylup-20(29)-ene (TTHL) [7]

The compound 3*β*,6*β*,16*β*- trihydroxylup-20(29)-ene (Fig. 1) was isolated and from the ethanolic extract of *C. leprosum* flowers and characterized according to the method described by Facundo et al. [7] using chromatography on a silica column, 1H and 13C NMR, mass spectrometry and an authentic sample for comparison. The lupane stock solution was prepared with ethanol P.A., and the concentration of the diluent was lower than 2 % of the reaction final volume.

### Ethics statement

The study was approved by the Research Ethics Committee of Centro de Pesquisas em Medicina Tropical – CEPEM under CAAE number: 02312812.2.0000.0011. The consent for participation in the study was obtained from the participants.

### Isolation of peripheral blood mononuclear cells (PBMC)

In order to obtain the PBMCs, blood from healthy volunteers, who had not used medication in the last 48 h, was collected into heparinized vacutainer tubes. PBMCs were isolated by density gradient using Histopaque 1077 (Sigma Aldrich), following the manufacturer’s instructions. Briefly, the blood diluted in phosphate-buffered saline (PBS) was layered on Histopaque in the ratio of 1:1 and subjected to centrifugation at 400 *xg* for 30 min at 4 °C. The white layer representing PBMCs was gently aspirated out and aseptically transferred into sterile centrifuge tubes. The suspended cells were then washed 3 times and cultured in a sterile RPMI assay medium {RPMI-1640 medium supplemented with 100 μg/mL of gentamicin, 2 mM of L-glutamine and 10 % fetal bovine serum (FBS)}. The number of cells was adjusted according to the amount of cells necessary for each experiment.

### MTT assay

The cytotoxicity of lupane against PBMCs was determined by an MTT assay [19], which analyzes the ability of living cells to reduce the 3-(4,5-dimethyl-2-thiazolyl)-2,5-diphenyl-2H-tetrazolium bromide (MTT) to a purple formazan product. Cells were plated in 96-well plates (2 × 10^5^/well) and maintained in RPMI assay medium (RPMI-1640 medium supplemented with 100 μg/mL of gentamicin, 2 mM of L-glutamine and 10 % FBS). PBMCs were incubated with RPMI (negative control), RPMI plus 2 % ethanol (diluent control) or different concentrations of lupane (0.3–12 μg/mL), diluted in ethanol, at 37 °C, under an atmosphere of 5 % CO_2_ for 1, 15 and 24 h. After incubation, the supernatant was replaced by a fresh medium containing MTT (0.5 μg/mL). Four hours later, the plates were washed 3 times with PBS, the formazan crystals were dissolved in 100 μL DMSO and the absorbance was measured with the Bio-Tek Synergy HT Multi-Detection (Winooski, VT) at 540 nm.

### Determination of interleukin-10 (IL-10) and tumor necrosis factor-α (TNF-α) production

PBMCs (2 × 10^5^/well) in an RPMI assay medium {RPMI-1640 medium supplemented with 100 μg/mL of gentamicin, 2 mM of L-glutamine and 10 % FBS) were incubated with lupane (0.3, 0.7 and 1.5 μg/mL) or without for 15 h at 37 °C, 5 % CO_2_. PBMCs were incubated with RPMI for the negative control, and with LPS (1 μg/mL) for the positive control. After incubation, plates were centrifuged at 405 *xg* for 5 min and the supernatants were collected for cytokine detection. The cytokines TNF-α and IL-10 were quantified using an enzyme immunoassay (EIA) kit (R&D Systems) according to the manufacturer’s instructions.

### Topoisomerase extraction from PBMCs

To test the activity of topoisomerase, the extraction was performed following the protocol provided on the TopoGEN site (www.topogen.com/html/enzyme_extracts.html). PBMCs at a density of 1 × 10^6^ cells were isolated as described in item 2.3 for the extraction of topoisomerases. Thus, PBMCs were resuspended in PBS and centrifuged at 800 *xg* for 3 min at 4 °C. From that moment, the whole procedure was performed on ice to prevent the action of proteases and inactivation of topoisomerase. Cells were resuspended in 5 mL of TEMP buffer (10 mM Tris–HCl, pH 7.5, 1 mM EDTA, 4 mM MgCl_2_, 0.5 mM PMSF) followed by centrifugation. The supernatant was discarded and 3 mL of cold TEMP buffer was added to the precipitate and placed on ice for 10 min. After this period, the tubes were centrifuged at 1500 *xg* for 10 min at 4 °C. The supernatant was discarded and 1 mL of cold TEMP buffer was added to the nuclear precipitate. The material was transferred into microtubes, and centrifuged again under the same conditions. The supernatant was then discarded and 100 mL of PE buffer (10 mM Tris–HCl, pH 7.5, 1 mM EDTA, 0.5 mM PMSF) was added with an equal volume of 1 M NaCl, homogenized and incubated in an ice bath for 30–60 min. Subsequently, the tubes were centrifuged at 14,000 *xg* for 15 min at 4 °C and the supernatant, containing topoisomerases I and II was used for the analysis of the enzyme activity.

### Topoisomerase I and II activity

The analyses of enzymatic activity of topoisomerases I and II were separately performed following the descriptions of TopoGEN Kit for each isoform.

For the analysis of TOPO I (topoisomerase I) activity, we used 3 μL of nuclear extract, 11 μL of deionized water, 2 μL of assay buffer, 1 μL of supercoiled DNA and 3 μL of lupane at 0.7, 1.5 or 6 μg/mL. For the control, 14 μL of deionized water was used. The experiment was conducted at 37 °C for 90 min, after which 5 μL of stop loading dye was added. The enzyme activity was verified in a 1 % agarose gel in TAE buffer, voltage 1–2.5 volt/cm. For the marker, we used the relaxed plasmid DNA and supercoiled DNA. After that, the gel was stained with 0.5 mg/mL of ethidium bromide at room temperature for 10 min and photographed (Loccus L- PIX Touch).

In order to assess TOPO II (topoisomerase II) activity, a volume of 3 μL of nuclear extract, 9.17 μL of deionized water, 4 μL of complete assay buffer 5X, 0.83 μL of kDNA (200 ng) and 3 μL of lupane at 0.7, 1.5 or 6 μg/mL. A volume of 12.17 μL of deionized water was used for the negative control and 3 μL of topoisomerase inhibitor (etoposide, 100 μM) was used for the positive control, followed by incubation at 37 °C for 90 min and the addition of 4 μL of stop loading dye. The enzyme activity was verified in a 1 % agarose gel in TAE buffer with ethidium bromide (0.5 μL/mL), with a voltage of 1–2.5 volt/cm. The kit provides decatened kDNA markers and linear kDNA which are also placed on the gel. After this process, the gel was photographed (Loccus L- PIX Touch).

### Molecular docking

As the first step to verify the toxicity of lupane, docking experiments between human topoisomerases I and II were performed. The PDB database was used in order to obtain the molecular structure of topoisomerase enzymes, and the AutoDock 4.0 [20] program was used for a rigid docking approach, that consists in fixing the distances and angles of the links of the biomolecular target and the binder during the calculation. The re-docking for TOPO I was done with Human TOPO I (1TL8) plus DNA plus indenoisoquinoline AI-III-52, based on data from Ioanoviciu et al. [21], since in this interaction, the compound in question had inhibitory activity on TOPO I . As for TOPO II, the model proposed by Wu et al. [22] using TOPO II (3QX3) plus DNA plus etoposide was chosen for the re-docking. After that, the cross-docking was processed according to the literature, using the enzyme TOPO I 1TL8 I or TOPOII 3QX3 plus double-stranded DNA plus lupane. Interactive visualization and comparative analysis of molecular structures were carried out in UCSF Chimera [23] and structure images with Persistence of Vision Raytracer (POV-ray) 3.62 (http://www.povray.org).

### Statistical analysis

Means and Standard error of the mean (S.E.M.) of viability and cytokine data were obtained and compared using one-way ANOVA, followed by the Bonferroni post-test in Graph-Pad PRISM 5.0. The differences were considered significant with probability levels of less than 0.05.

## Results

### *Evaluation of* cytotoxicity

**Fig. 2:**
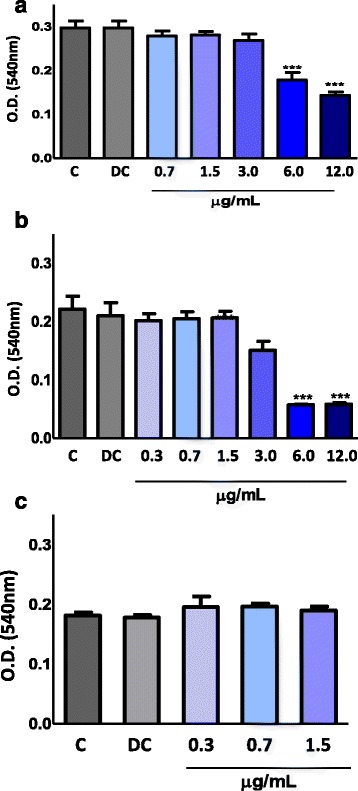
Effect of lupane on PBMC viability after 1 h (**a**), 15 h (**b**) and 24 h (**c**) of incubation. Peripheral human mononuclear cells (PBMCs) were isolated from buffy coats of healthy adult blood donors through a density gradient method. The cells of a density of 2×10^5^ cells were incubated with different concentrations of lupane or RPMI (negative control) or RPMI plus 2 % ethanol (diluent control) at 37 °C in a humidified atmosphere of 5 % CO_2_. After that, PBMC viability was assessed by the MTT method. Values represent the mean ± S.E.M. from 4–5 donors ***P < 0.001 compared with the control (ANOVA)

PBMCs were treated with lupane (0.3–12 μg/mL) to test the toxic effect of this compound on mammalian cells. After 1, 15 and 24 h of incubation, cell viability was evaluated by the MTT assay. Lupane showed no cytotoxic effect at the concentration range between 0.3 and 1.5 μg/mL at all incubation periods (Fig. 2a, b and c). However, at 12 and 6 μg/mL, this compound seemed to be toxic to the cells at 1 h of incubation, resulting in the death of 52 % and 40 % of the cells, respectively, when compared to the control (Fig. 2a). After 15 h of incubation, cell death at these concentrations was 74 %, whereas at the concentration of 3 μg/mL of lupane, death was 32 % and the LC_50_ at this period was about 4.3 μg/mL (Fig. 2b). Ethanol, used to dilute the compound, showed no cytotoxicity to the cells.

### Evaluation of TNF-α and IL-10 concentrations

**Fig. 3:**
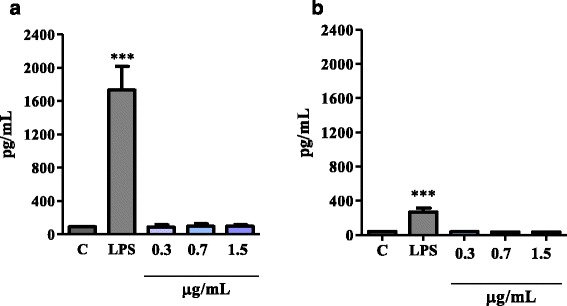
Effect of lupane on the release of TNF-α (**a**) and IL-10 (**b**) by PBMCs. PBMCs (2×10^5^) were incubated with lupane at 0.3, 0.7 and 1.5 μg/mL or RPMI (negative control) or LPS (positive control, 1 μg/mL) at 37 °C in a humidified atmosphere of 5 % CO_2_ for 15 h. The concentrations of TNF-α and IL-10 in the supernatant were quantified by a specific Enzyme Immune Assay (EIA). The results were expressed in pg/mL of TNF-α or IL-10 and represent the mean ± S.E.M. of four donors. ***P < 0.001 compared to control (ANOVA)

In order to verify the ability of lupane to activate PBMC cellular functions and stimulate the pro-inflammatory or anti-inflammatory cytokine release, such as TNF-α and IL-10, respectively, the cells were incubated with non-cytotoxic concentrations of lupane or RPMI (negative control) or LPS (1 μg/mL, positive control). As shown in Fig. 3, LPS, a positive control, induced a significant release of TNF-α (A) and IL-10 (B) when compared to RPMI, a negative control. On the other hand, lupane, at all concentrations (0.3 up to 1.5 μg/mL), did not induce TNF-α and IL-10 production.

### Topoisomerase I and II activity

**Fig. 4:**
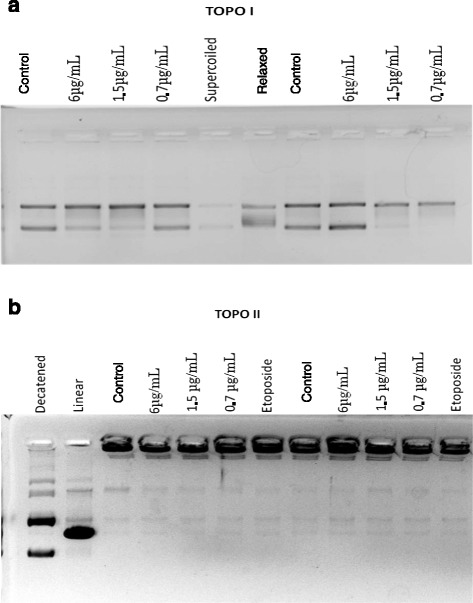
Effect of lupane on topoisomerases I and II activity. Electrophoresis in 1 % agarose gel to assess the activity of TOPO I (**a**): nuclear extracts of PBMCs and supercoiled DNA (TopoGen) (control), lupane (6; 1.5 or 0.7 μg/mL plus nuclear extract of PBMCs and supercoiled DNA; markers (relaxed and supercoiled DNA). (Representatives of 2n). Electrophoresis in 1 % agarose gel with ethidium bromide to assess the activity of TOPO II. **b**: nuclear extract of PBMCs and kDNA (TopoGen) (control), lupane (6; 1.5 or 0.7 μg/mL plus nuclear extract of PBMCs plus kDNA, nuclear extract of PBMCs plus kDNA plus etoposide (inhibitor); markers (linear and decatenated kDNA). (Representatives of 2n)

To assess lupane’s effect on topoisomerase I (TOPOI), the TopoGen^®^ kit was used in order to detect the enzyme action capacity on a supercoiled DNA substrate. To visualize the enzyme action, cell nuclear extract was used as the positive control. As shown in Fig. 4, the results obtained showed that the TOPO I enzyme has activity since most of the DNA is in linear form in the control and lupane groups.

For the analysis of lupane’s effect on topoisomerase II (TOPO II) enzyme activity, the TopoGen^®^ kit was also used. In addition to the lupane and control groups, one more group was inserted, an etoposide group, a TOPO II inhibitor. According to Fig. 4, the results obtained showed that lupane did not interfere in the enzyme activity since DNA was cleaved. Moreover, it could also be observed that the lupane group and the control group had the same pattern on the gel.

### Molecular docking

**Fig. 5:**
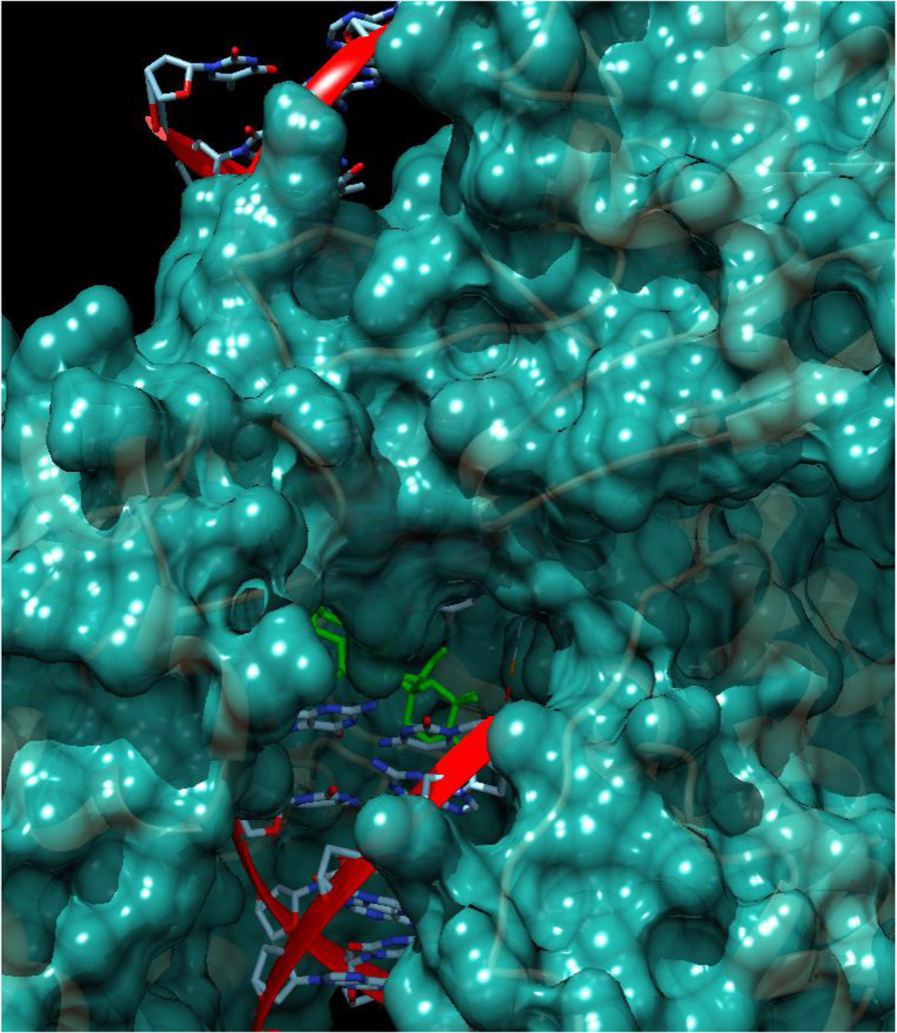
Molecular Docking of lupane in Topoisomerase I with DNA (PDB: 1TL8). The binding energy resulted in −8.86 kcal/mol. The lupane is shown in green, DNA helix is shown in red ribbon and the enzyme is shown in cyan surface

**Fig. 6:**
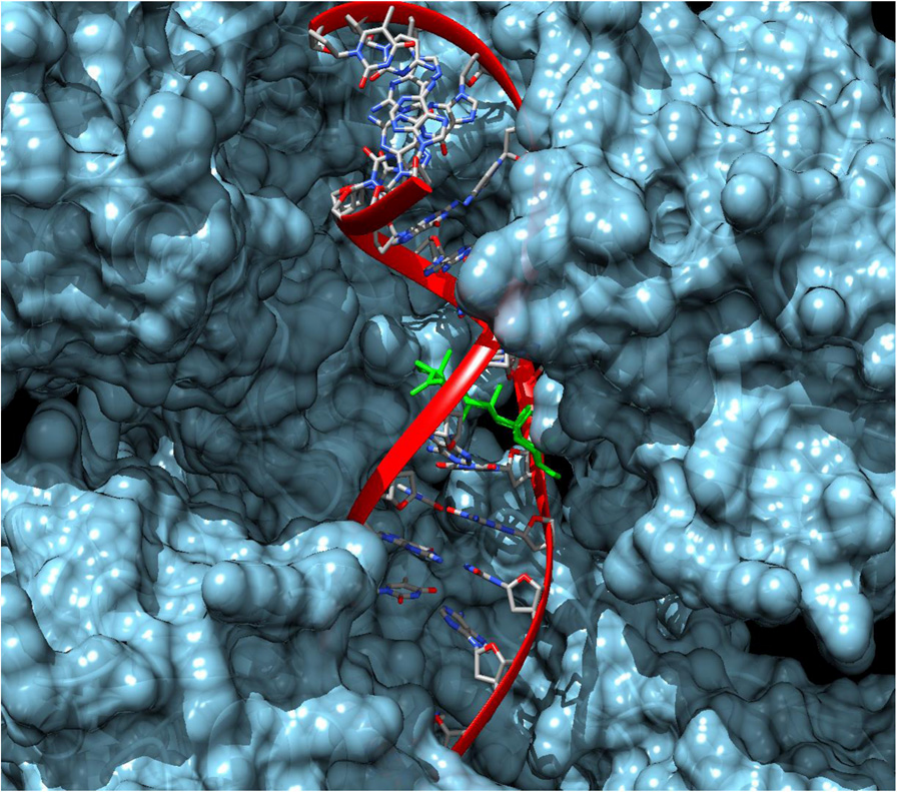
Molecular Docking of lupane in Topoisomerase II with DNA (3QX3). The binding energy resulted in −9.91 kcal/mol. The lupane is shown in green, DNA helix is shown in red ribbon and the enzyme is shown in light blue surface

The molecular cross-docking of TOPO I, in which one molecule of lupane, DNA and one molecule of enzyme were used, was rigid. As such, the interaction resulted in −8.86 kcal/mol of energy (Fig. 5). However, the interaction between DNA + TOPO I and the known TOPO I inhibitor, indenoisoquinoline AI-III-52 [21], resulted in −10.35 kcal/mol of energy. Accordingly, we concluded that lupane is not an efficient TOPO I inhibitor. On the other hand, the procedure of re-docking performed with TOPO II, a double-stranded molecule of DNA and the inhibitor etoposide, showed −12.81 kcal/mol of energy [22]. The cross-docking of the same complex, using lupane instead of etoposide resulted in −9.91 kcal/mol of energy in its most stable configuration (Fig. 6). As such, we concluded that lupane has no inhibitory activity on TOPO II.

## Discussion

Studies involving species of the genus *Combretum* are constantly increasing and various compounds with known biological properties, such as tannins, flavonoids, saponins, coumarins, triterpenes, derivatives of ellagic acid, antracenics glycosides, and derivatives of phenanthrene have already been isolated and identified. These compounds are probably responsible for the biological properties proven in scientific research [24].

Triterpene lupane 3*β*, 6*β*, 16*β*-trihydroxylup-20(29)-ene (TTHL) was isolated from *C. leprosum* Mart. in 1993 by Facundo et al., and until today its biological properties haven’t been fully established. Thus, studies focusing on clarifying these properties are of paramount importance.

Triterpene compounds are found in several plant species, and some compounds of this group seem to exert important biological activities such as anti- inflammatory, anticancer and antibacterial ones [25]. They are constituted by isoprene units and may be divided into derivatives of squalene, tetracyclic and pentacyclic types [26]. The pentacyclic lupane type of triterpenoids represents one of the very important classes of natural products. The compounds of this class, which include betulin and betulinic acid, show significant antitumor activity on different types of cancers [27, 28]. Several studies showed that lupeol, betulin, betulinic acid, oleanolic and ursolic acid are multitarget agents, that had differences in their efficacy in several assays [28–38].

In this study, the cytotoxicity on human PBMCs was conducted at three different times (1, 15 and 24 h). The results showed that the triterpene lupane had greater toxicity when it remained in contact with the PBMCs for a longer time. Therefore, low concentrations of lupane were used to assess cell function, since these concentrations did not change the cell viability.

Cisplatin, one of the most effective anticancer drug, is a cytotoxic agent, which kills a variety of cancer cells by damaging DNA [39, 40]. Depending on the cell type and concentration, cisplatin induces cytotoxicity by interference with transcription and/or DNA replication mechanisms [41–43]. During therapy with cisplatin, this toxicity is cumulative and irreversible, so fractional or metronomic dosing schedules significantly reduce its toxicity [44, 45]. Furthermore, cisplatin has been used in combination with other drugs in order to improve the antiproliferative activity of both compounds. Emmerich et al. [46] synthesized several lupane-type triterpenoid derivatives, such as betulinic acid, conjugated with cisplatin and showed that the double drugs were less cytotoxic than the precursors. It is important to note that the only report about the effect of lupane isolated from *C. leprosum* on cell toxicity was performed with mice peritoneal elicited macrophages. The study demonstrated that 5 μg/mL of lupane showed no toxicity on this cell after 24 h of incubation. However, both crude extract and isolated lupane (5 μg/mL) presented potent toxic activity against promastigotes of *L. amazonensis* [13]. In a recent study, it became clear that lupane is also effective in eliminating intracellular amastigotes, without affecting the viability of the host cells. Teles et al. [47] showed that there was a reduction in the rate of infected macrophages and in the number of intracellular parasites from 24 h of treatment. Because of the changes observed, it was suggested that lupane might interfere in the action of enzymes important for the maintenance of kinetoplast or mitochondrial structure.

Topoisomerases catalyze topological changes in DNA strands during DNA replication, transcription, recombination and DNA repair [48, 49]. Mammalian somatic cells have six genes of DNA topoisomerase (TOP): two topoisomerases I TOP I (TOP I and mitochondrial TOP I), two topoisomerases II TOP II (TOP IIα and IIβ) and two topoisomerases III (TOP IIIα and IIIβ) [50, 51]. Topoisomerases are classified depending on the number of DNA strands cut in one round of action: cutting a strand of DNA, DNA-topoisomerases of type I (TOPO I) or cleavage of both strands of the target DNA, DNA-topoisomerase type II (TOPO II).

Human TOPO I is a nuclear phosphoprotein with 765 amino acids and a molecular weight of 91 kDa [52], which is constitutively expressed throughout the cell cycle [53, 54]. This enzyme shows a diffuse pattern of distribution in the nucleoplasm with accumulation in the nucleolus [55]. The catalytic activity of TOPO I is specifically inhibited by camptothecin, an alkaloid of *Camptotheca acuminata* and similar drugs, such as topotecan (Hycantina®, GlaxoSmithKline) [56, 57] and irinotecan (Camptosar, Pfizer) [58, 59]. Camptothecin has powerful anti-tumor activity by inhibiting synthesis of nucleic acids and inducing breaks in DNA molecules ([60]).

Topo II has two isoforms: α and β isoforms [61], that share ATPase activity of the N-terminal domain and the central domain containing the catalytic tyrosine (Tyr804 in the α isoform and Tyr821 in β). The C-terminal domain gives the functional specificity of the two isoforms [62, 63]. Despite having a three-dimensional structure similar to that of the α isoform, the β isoform dimer has less sensitivity to most anti-topoisomerase II drugs *in vitro* and *in vivo* [64, 65].

During cell proliferation, topoisomerases participate in the maintenance and replication of DNA. Moreover, in tumor tissues, the expression of TOPO I and II is greater than cells with normal metabolism [66]. Therefore, drugs that act on these enzymes are used in the treatment of patients with several types of cancer. Currently, transcription inhibitors are under investigation as potential anti-cancer agents [67].

The results of this study showed that the compound studied does not interfere in the activity of topoisomerases isolated from human PBMCs, even in the concentration of 6 μg/mL, which is cytotoxic. Moreover, the molecular docking studies showed little or no probability of human topoisomerase enzyme inhibition by lupane, confirming the results obtained in biological assays. On the other hand, Teles et al. [47] showed by bioinformatic analysis that lupane has a high binding affinity with topoisomerase IB of *Leishmania braziliensis*. The trypanosomatids topoisomerase I are heterodimeric and located in the nucleus, associated with the genomic DNA and in the kinetoplast associated with kDNA [68–70]. It is important to mention that DNA topoisomerases as a potential drug target for human pathogenic trypanosomatids is based on the clinical trials of camptothecin derivatives in anticancer therapy [70]. *Leishmania* topoisomerase I differ from its orthologous proteins in other eukaryotic cells. While the DNA topoisomerases I from mammalian hosts are monomeric enzymes, the trypanosomatid type I DNA topoisomerases are heterodimeric [70, 71]. The amino acid substitutions and significant structural differences between human and parasite topoisomerases make these enzymes such molecular targets of interest for chemotherapeutic intervention in diseases caused by trypanosomatid parasites.

Based on preclinical results obtained in this study, we highlight that the compound studied (lupane) has moderate cytotoxicity, does not induce the production of TNF-α and IL-10, and does not act on human topoisomerases. Taking into account the results of this study and the reports about the anti-inflammatory and leishmanicidal activity of the 3*β*, 6*β*, 16*β*-trihydroxylup-20(29)-ene lupane, we suggest that this compound may serve as a biotechnological tool in the future.

## References

[1] Agra MF, Freitas PF, Barbosa-Filho JM (2007). Synopsis of the plants known as medicinal and poisonous in Northeast of Brazil. Rev Bras Farmacogn.

[2] Hoareau L, Da Silva EJ (1999). Medicinal plants: A re-emerging health aid. Eletronic J. Biotechnol.

[3] Sneader W (1996). Drug Prototypes and Their Exploitation.

[4] Calderon LA, Silva-Jardim I, Zuliani JP, Silva AA, Ciancaglini P, Pereira-da-Silva LH (2009). Amazonian biodiversity: a view of drug development for Leishmaniasis and Malaria. J Braz Chem Soc.

[5] Tan F, Shi S, Zhong Y, Gong X, Wang Y (2002). Phylogenetic relationships of Combretoideae (Combretaceae) inferred from plastid, nuclear gene and spacer sequences. Journal of Plant Research.

[6] Pettit GR, Singh SB, Boyd MR, Hamel E, Pettit RK, Schmidt JM (1995). Antineoplastic agents. 291. Isolation and synthesis of combretastatins A-4, A-5, and A-6(1a). Med Chem.

[7] Facundo VA, Andrade CHS, Silveira ER, Braz-Filho R, Hufford C (1993). Triterpenes and flavonoids from *Combretum leprosum*. Phytochesmistry.

[8] Pio Correa M (1984). Diccionário de Plantas Úteis do Brasil e das Exóticas Cultivadas.

[9] Lira SRD, Almeida RN, Almeida FRC, Oliveira FS, Duarte JC (2002). Preliminary studies on the analgesic properties of the ethanol extract of *C. leprosum*. Pharm Biol.

[10] Pietrovsky EF, Rosa KA, Facundo VA, Rios K, Marques MCA, Santos ARS (2006). Antinociceptive properties of the ethanolic extract and of the triterpene 3β, 6β, 16β-trihidroxilup-20(29)-ene obtained from the flowers of *Combretum leprosum* in mice. Pharmacology, Biochemistry and Behavior.

[11] Fernandes FF, Tomaz MA, El-Kik CZ, Monteiro-Machado M, Strauch MA, Cons BL (2014). Counteraction of *Bothrops* snake venoms by *Combretum leprosum* root extract and arjunolic acid. J Ethnopharmacol.

[12] Evaristo FF, Albuquerque MR, Dos Santos HS, Bandeira PN, Avila Fdo N, Da Silva R (2014). Antimicrobial effect of the triterpene 3β,6β,16β-trihydroxylup-20(29)-ene on planktonic cells and biofilms from Gram positive and Gram negative bacteria. Biomed Res Int.

[13] Teles CBG, Moreira LS, Silva AAE, Facundo VA, Zuliani JP, Stábeli RG (2011). Activity of the Lupane isolated from *Combretum leprosum* against *Leishmania amazonensis* promastigotes. J Braz Chem Soc.

[14] Nagase M, Oto J, Sugiyama S, Yube K, Takaishi Y, Sakato N (2003). Apoptosis induction in HL-60 cells an inhibition of topoisomerase II by triterpene celastrol. Biosci Biotechnol. Biochem.

[15] Sethi G, Ahn KS, Pandey MK, Aggarwal BB (2007). Celastrol, a novel triterpene, potentiates TNF-induced apoptosis and suppresses invasion of tumor cells by inhibiting NF-kappaB-regulated gene products and TAK-1-mediated NF-kappaB activation. Blood.

[16] Wang P, Ownby S, Zhang Z, Yuan W, Li S (2010). Cytotoxicity and inhibiton of DNA topoisomerase I of polyhydroxyleted triterpenoids and triterpenoid glycosides. Chem lett.

[17] Chowdhury AR, Mandal S, Goswami A, Ghosh M, Mandal L, Chakraborty D (2003). Dihydrobetulinic acid induces apoptosis in *Leishmania donovani* by targeting DNA topoisomerase I and II: implications in antileishmanial therapy. Mol Med.

[18] Chowdhury S, Mukherjee T, Sengupta S, Chowdhury SR, Mukhopadhyay S, Majumder HK (2011). Novel betulin derivatives as antileishmanial agents with mode of action targeting type IB DNA topoisomerase. Mol Pharmacol.

[19] Reilly TP, Bellevue FH, Woster PM (1998). Compararion of the in vitro citotoxicity os hydroxylamine metabolites of sulfamethoxazole and dapsone. Biochem Pharmacol.

[20] Morris GM, Huey R, Lindstrom W, Sanner MF, Belew RK, Goodsell DS (2009). AutoDock4 and AutoDockTools4: Automated docking with selective receptor flexibility. J Comp Chem.

[21] Ioanoviciu A, Antony S, Pommier Y, Staker BL, Stewart L, Cushman M (2005). Synthesis and mechanism of action studies of a series of norindenoisoquinoline topoisomerase I poisons reveal an inhibitor with a flipped orientation in the ternary DNA-enzyme-inhibitor complex as determined by X-ray crystallographic analysis. J Med Chem.

[22] Wu CC, Li TK, Farh L, Lin LY, Lin TS, Yu YJ (2011). Structural basis of type II topoisomerase inhibition by the anticancer drug etoposide. Science.

[23] Goddard TD, Huang CC, Couch GS, Greenblatt DM, Meng EC, Ferrin TE (2004). UCSF Chimera - a visualization system for exploratory research and analysis. J Comput Chem.

[24] Morais-Lima GR, De Sales IR, Caldas-Filho MR, De Jesus NZ, De Sousa FH, Barbosa-Filho JM (2012). Bioactivities of the genus Combretum (Combretaceae): a review. Molecules.

[25] Jesus JA, Lago JH, Laurenti MD, Yamamoto ES, Passero LF (2015). Antimicrobial activity of oleanolic and ursolic acids: an update. Evid Based Complement Alternat Med..

[26] Thimmappa R, Geisler K, Louveau T, O’Maille P, Osbourn A (2014). Triterpene biosynthesis in plants. Annu Rev Plant Biol.

[27] Kommera H, Kaluđerović GN, Kalbitz J, Paschke R (2011). Lupane triterpenoids--betulin and betulinic acid derivatives induce apoptosis in tumor cells. Invest New Drugs.

[28] Laszczyk MN (2009). Pentacyclic triterpenes of the lupane, oleanane and ursane group as tools in cancer therapy. Planta Med.

[29] Baltina LA, Flekhter OB, Nigmatullina LR, Boreko EI, Pavlova NI, Nikolaeva SN (2003). Lupane triterpenes and derivatives with antiviral activity. Bioorg Med Chem Lett.

[30] De Silva ML, David JP, Silva LC, Santos RA, David JM, Lima LS (2012). Bioactive oleanane, lupane and ursane triterpene acid derivatives. Molecules.

[31] Fulda S (2009). Betulinic acid: A natural product with anticancer activity. Mol Nutr Food Res.

[32] Fulda S, Friesen C, Los M, Scaffidi C, Mier W, Benedict M (1997). Betulinic acid triggers CD95 (APO-1/Fas)- and p53-independent apoptosis via activation of caspases in neuroectodermal tumors. Cancer Res.

[33] Fulda S, Debatin KM (2000). Betulinic acid induces apoptosis through a direct effect on mitochondria in neuroectodermal tumors. Med Pediatr Oncol.

[34] Galaĭko NV, Tolmacheva IA, Grishko VV, Volkova LV, Prevozchikova EN, Pestereva SA (2010). Antiviral activity of 2, 3-secotriterpenic hydrazones of lupane and 19 beta, 28-epoxy-18 alpha-oleanane type. Bioorganicheskaya Khimiya.

[35] Kwon HJ, Shim JS, Kim JH, Cho HY, Yum YN, Kim SH (2002). Betulinic acid inhibits growth factor-induced *in vitro* angiogenesis via the modulation of mitochondrial function in endothelial cells. Jpn J Cancer Res.

[36] Liaw CC, Chen YC, Huang GJ, Tsai YC, Chien SC, Wu JH (2013). Anti-inflammatory lanostanoids and lactone derivatives from *Antrodia camphorata*. J Nat Prod.

[37] Melzig MF, Bormann H (1998). Betulinic acid inhibits aminopeptidase N activity. Planta Med.

[38] Mokoka TA, McGaw LJ, Mdee LK, Bagla VP, Iwalewa EO, Eloff JN. Antimicrobial activity and cytotoxicity of triterpenes isolated from leaves of Maytenus undata (Celastraceae). BMC Complement. Altern. Med. 2013, 13, doi:10.1186/1472-6882-13-111 10.1186/1472-6882-13-111 PMC3711988 23688235

[39] Basu A, Krishnamurthy S (2010). Cellular responses to Cisplatin-induced DNA damage. J Nucleic Acids.

[40] Sedletska Y, Giraud-Panis MJ, Malinge JM (2005). Cisplatin is a DNA-damaging antitumour compound triggering multifactorial biochemical responses in cancer cells: importance of apoptotic pathways. Current Medicinal Chemistry Anti-Cancer Agents.

[41] Florea AM, Büsselberg D (2011). Cisplatin as an anti-tumor drug: cellular mechanisms of activity, drug resistance and induced side effects. Cancers (Basel).

[42] Rademaker-Lakhai JM, Crul M, Zuur L, Baas P, Beijnen JH, Simis YJ (2006). Relationship between cisplatin administration and the development of ototoxicity. J Clin Oncol.

[43] Tsang RY, Al-Fayea T, Au HJ (2009). Cisplatin overdose: Toxicities and management. Drug Saf.

[44] Hartmann JT, Kollmannsberger C, Kanz L, Bokemeyer C (1999). Platinum organ toxicity and possible prevention in patients with testicular cancer. Int J Cancer.

[45] Shen FZ, Wang J, Liang J, Mu K, Hou JY, Wang YT (2010). Low-dose metronomic chemotherapy with cisplatin: can it suppress angiogenesis in H22 hepatocarcinoma cells?. Int J Exp Pathol.

[46] Emmerich D, Vanchanagiri K, Baratto LC, Schmidt H, Paschke R (2014). Synthesis and studies of anticancer properties of lupane-type triterpenoid derivatives containing a cisplatin fragment. Eur J Med Chem.

[47] Teles CB, Moreira-Dill LS, Silva A de A, Facundo VA, de Azevedo WF, da Silva LH (2015). A lupane-triterpene isolated from *Combretum leprosum* Mart. fruit extracts that interferes with the intracellular development of *Leishmania* (L.) *amazonensis in vitro*. BMC Complement Altern Med.

[48] Koster DA, Croquette V, Dekker C, Shuman S, Dekker NH (2005). Friction band torque govern the relaxation of DNA supercoils by eukaryotic topoisomerase IB. Nature.

[49] Liu LF, Liu CC, Alberts BM (1980). Type II DNA topoisomerases: enzymes that can unknot a topologically knotted DNA molecule via a reversible double-strand break. Cell.

[50] Champoux JJ (2001). DNA topoisomerases: structure, function, and mechanism. Annu Rev Biochem.

[51] Wang JC (2002). Cellular roles of DNA topoisomerases: a molecular perspective. Nat Rev Mol Cell Biol.

[52] D’Arpa P, Machlin PS, Ratrie H, Rothfield NF, Cleveland DW, Earnshaw WC (1988). cDNA cloning of human DNA topoisomerase I: catalytic activity of a 67.7-kDa carboxyl-terminal fragment. Proc Natl Acad Sci USA.

[53] Baker SD, Wadkins RM, Stewart CF, Beck WT, Danks MK (1995). Cell cycle analysis of amount and distribution of nuclear DNA topoisomerase I as determined by fluorescence digital imaging microscopy. Cytometry.

[54] Meyer KN, Kjeldsen E, Straub T, Knudsen BR, Hickson ID, Kikuchi A (1997). Cell cycle-coupled relocation of types I and II topoisomerases and modulation of catalytic enzyme activities. J Cell Biol.

[55] Christensen MO, Barthelmes HU, Feineis S, Knudsen BR, Andersen AH, Boege F (2002). Changes in mobility account for camptothecin-induced subnuclear relocation of topoisomerase I. J Biol Chem.

[56] Beldner MA, Sherman CA, Green MR, Garrett-Mayer E, Chaudhary U, Meyer ML (2007). Phase I dose escalation study of vinorelbine and topotecan combination chemotherapy in patients with recurrent lung cancer. BMC Cancer.

[57] Lim WT, Baggstrom MQ, Read W, Fracasso PM, Govindan R (2008). A Phase I trial of weekly docetaxel and topotecan for solid tumors. Acta Oncol.

[58] Friedman HS, Keir ST, Houghton PJ (2003). The emerging role of irinotecan (CPT-11) in the treatment of malignant glioma in brain tumors. Cancer.

[59] Han JY, Lim HS, Park YH, Lee SY, Lee JS (2009). Integrated pharmacogenetic prediction of irinotecan pharmacokinetics and toxicity in patients with advanced non-small cell lung cancer. Lung Cancer.

[60] Hsiang YH, Hertzberg R, Hecht S, Liu LF (1985). Camptothecin induces protein-linked DNA breaks via mammalian DNA topoisomerase I. J Biol Chem.

[61] Drake FH, Zimmerman JP, McCabe FL, Bartus HF, Per SR, Sullivan DM (1987). Purification of topoisomerase II from Amsacrine-resistant P388 leukemia cells. Evidence for two forms of the enzyme. J Biol Chem.

[62] Krieser RJ, Maclea KS, Park JP, Eastman A (2001). The cloning, genomic structure, localization, and expression of human deoxyribonuclease IIbeta. Gene.

[63] Linka RM, Porter AC, Volkov A, Mielke C, Boege F, Christensen MO (2007). C-terminal regions of topoisomerase IIalpha and IIbeta determine isoform-specific functioning of the enzymes *in vivo*. Nucleic Acids Res.

[64] Asano T, Kleinerman ES, Zwelling LA, Zhou Z, Fukunaga Y (2005). Adenovirus-mediated human topoisomerase IIalpha gene transfer increases the sensitivity of etoposide-resistant human and mouse breast cancer cells. Acta Oncol.

[65] Austin CA, Marsh KL (1998). Eukaryotic DNA topoisomerase II beta. Bioessays.

[66] Kellner U, Rudolph P, Parwaresch R (2000). Human DNA-topoisomerase – diagnostic and therapeutic implications for cancer. Onkologie.

[67] Stellrecht CM, Chen LS (2011). Transcription inhibition as a therapeutic target for cancer. Cancers.

[68] Bodley AL, Chakraborty AK, Xie S, Burri C, Shapiro TA (2003). An unusual type IB topoisomerase from African trypanosomes. Proc Natl Acad Sci USA.

[69] Das BB, Sen N, Ganguly A, Majumder HK (2004). Reconstitution and functional characterization of the unusual bi-subunit type I DNA topoisomerase from *Leishmania donovani*. FEBS Lett.

[70] Reguera RM, Redondo CM, Gutierrez De Prado R, Pérez-Pertejo Y, Balaña-Fouce R (2006). DNA topoisomerase I from parasitic protozoa: a potential target for chemotherapy. Biochim Biophys Acta.

[71] Villa H, Otero Marcos AR, Reguera RM, Balaña-Fouce R, García-Estrada C, Pérez-Pertejo Y (2003). A novel active DNA topoisomerase I in *Leishmania donovani*. J Biol Chem.

